# Qualitative Evaluation of mHealth Implementation for Infectious Disease Care in Low- and Middle-Income Countries: Narrative Review

**DOI:** 10.2196/55189

**Published:** 2024-12-13

**Authors:** Josephine Greenall-Ota, H Manisha Yapa, Greg J Fox, Joel Negin

**Affiliations:** 1 Faculty of Science, University of Sydney, Sydney, NSW, Australia; 2 Sydney Infectious Diseases Institute, Faculty of Medicine and Health, University of Sydney, Science Rd, Sydney, NSW, 2050, Australia, 61 2 9351 2222; 3 Westmead Hospital, Western Sydney Local Health District, Westmead, Sydney, NSW, Australia; 4 Faculty of Medicine and Health, University of Sydney, Sydney, NSW, Australia; 5 Woolcock Institute of Medical Research, Macquarie Park, Sydney, NSW, Australia; 6 Royal Prince Alfred Hospital, Sydney Local Health District, Camperdown, Sydney, NSW, Australia; 7 School of Public Health, Faculty of Medicine & Health, University of Sydney, Sydney, NSW, Australia

**Keywords:** mHealth, implementation, LMIC, infectious diseases, Tailored Implementation for Chronic Diseases, mobile phone, interventions, short messaging service, chronic disease, narrative review, implementation, barrier, mHealth intervention, infectious disease, screening, community, design, health system, SMS, app

## Abstract

**Background:**

Mobile health (mHealth) interventions have the potential to improve health outcomes in low- and middle-income countries (LMICs) by aiding health workers to strengthen service delivery, as well as by helping patients and communities manage and prevent diseases. It is crucial to understand how best to implement mHealth within already burdened health services to maximally improve health outcomes and sustain the intervention in LMICs.

**Objective:**

We aimed to identify key barriers to and facilitators of the implementation of mHealth interventions for infectious diseases in LMICs, drawing on a health systems analysis framework.

**Methods:**

We followed the PRISMA (Preferred Reporting Items for Systematic Reviews and Meta-Analyses) checklist to select qualitative or mixed methods studies reporting on determinants of already implemented infectious disease mHealth interventions in LMICs. We searched MEDLINE, Embase, PubMed, CINAHL, the Social Sciences Citation Index, and Global Health. We extracted characteristics of the mHealth interventions and implementation experiences, then conducted an analysis of determinants using the Tailored Implementation for Chronic Diseases framework.

**Results:**

We identified 10,494 titles for screening, among which 20 studies met our eligibility criteria. Of these, 9 studies examined mHealth smartphone apps and 11 examined SMS text messaging interventions. The interventions addressed HIV (n=7), malaria (n=4), tuberculosis (n=4), pneumonia (n=2), dengue (n=1), human papillomavirus (n=1), COVID-19 (n=1), and respiratory illnesses or childhood infectious diseases (n=2), with 2 studies addressing multiple diseases. Within these studies, 10 interventions were intended for use by health workers and the remainder targeted patients, at-risk individuals, or community members. Access to reliable technological resources, familiarity with technology, and training and support were key determinants of implementation. Additional themes included users forgetting to use the mHealth interventions and mHealth intervention designs affecting ease of use.

**Conclusions:**

Acceptance of the intervention and the capacity of existing health care system infrastructure and resources are 2 key factors affecting the implementation of mHealth interventions. Understanding the interaction between mHealth interventions, their implementation, and health systems will improve their uptake in LMICs.

## Introduction

Mobile health (mHealth) technologies, defined by the World Health Organization (WHO) as “the use of mobile and wireless technologies to support health objectives,” have the potential to improve health outcomes globally, including in low- and middle-income countries (LMICs) [1-6]. This is achievable through improving patient education, improving disease self-management, decreasing health care costs, and performing remote monitoring of patients, as reported in a recent systematic review of mHealth in LMICs [3]. In addition, mHealth can support preventative measures, facilitate disease management, or support health workers to strengthen the delivery of health care [7-10]. The WHO has highlighted the need to advance national digital health strategies that can facilitate universal health care [11]. The WHO’s “Recommendations on digital interventions for health system strengthening” highlights that digital technologies, including mHealth, can directly address health system challenges by supporting more widespread coverage across population groups and improving the quality and affordability of health care [6]. This digital transformation of the health care system has been made possible by the widespread availability of affordable digital technology; currently, 95% of the world population has internet access [12].

mHealth interventions targeting infectious diseases care have the potential to greatly transform the health care landscape of LMICs, where infectious diseases still represent a substantial burden [13,14]. This is particularly important given health system challenges such as low health service utilization, poor adherence to clinical protocols among health workers, and geographic inaccessibility of health facilities [6].

The success and sustainability of mHealth interventions require overcoming context-specific barriers and enhancing facilitators of mHealth implementation; these factors must be considered prior to intervention design. The WHO’s “Global Strategy on Digital Health 2020‐2025” acknowledged the need to adapt digital health intervention implementation to unique national contexts, health situations, and trends, as well as a country’s vision, available resources, and core values [11]. The WHO’s “Recommendations on digital interventions for health system strengthening” further identified key implementation enablers including health content aligning with recommended practices, intervention functionality, and greater leadership and governance [6]. The WHO has emphasized that recognizing and addressing digital health implementation challenges uniquely faced by the least-developed countries is a large factor influencing the scalability and sustainability of emerging mHealth technologies [11].

mHealth interventions in LMICs have had limited success due to a range of health system factors not considered during the development and implementation of interventions [3-5,15-17]. Many mHealth interventions in LMICs remain as pilot studies that investigate feasibility, usability, and effectiveness, and they have not been scaled-up for integration within the broader health care system [17,18]. mHealth initiatives have often been developed for use in higher-resource health systems, with little consideration of differing contexts affecting implementation, such as social norms around a health-promoting behavior or access to resources [17]. Industry representatives, such as those from mobile phone providers, often push the scale-up of mHealth interventions rather than researchers, governments, or health workers [19]. This excludes crucial end user perspectives when developing mHealth interventions and risks having market-driven motives unrelated to health care encouraging the scale-up of interventions [19]. mHealth intervention teams also often fail to understand the relationship between users and mHealth technologies [2,19-21]. Previously reported barriers to the widespread adoption of mobile technologies that uniquely concern LMICs include poor mobile network coverage, limited health care workforce capacity, limited data access, or negative health worker and patient perceptions toward mHealth interventions [3-5,15-17].

Among the limited number of published reviews evaluating mHealth implementation, there is a lack of rigorous evaluation regarding the design and implementation of mHealth interventions to aid policymakers [3,16,20,22,23].

We therefore conducted a narrative review of the existing literature to understand the determinants of mHealth implementation for infectious diseases in LMICs. The review aims to consider the broader context, drawing on a comprehensive health systems analysis framework.

## Methods

We searched MEDLINE, Embase, PubMed, CINAHL, the Social Sciences Citation Index, and Global Health. We collected studies that were the earliest available indexed in the above databases, up to and including May 31, 2023; the studies were exclusively in English.

### Selection Criteria

#### Inclusion Criteria

1. Study population: We included individuals (of any age) with infectious diseases in LMICs. We took LMIC search terms from the Cochrane Effective Practice and Organisation of Care LMIC filters, defined according to the World Bank Classification (2022) [24]. We did not restrict the type of participants in the intervention (ie, we included health workers, patients, carers, general community members, and multiple types of participants).
2. Intervention: We defined mHealth interventions as per the WHO [6]. These interventions included SMS, electronic decision-support tools, educational tools, apps, and other strategies to improve health care delivery. We included interventions that used either mobile phones, smartphones, or tablet devices, conducted at any level of the health care system.
3. Comparator: We included studies where the current standard of care was a comparator, where applicable, in addition to studies without a control group listed.
4. Outcomes: We included qualitative and mixed methods studies that included a description of the mHealth intervention and implementation processes and reported on factors affecting implementation (eg, acceptability, feasibility, essential resources) based on interviews or discussion groups.

#### Exclusion Criteria

We excluded formative research studies (ie, studies conducted before fully developing or implementing an intervention); study protocols; interventions involving computers or web-based health care (eg, websites); telehealth interventions (defined as consultation with a health worker via a mobile phone either through SMS or phone calls); and quantitative studies including randomized controlled trials, Likert scale surveys, and impact evaluations, as they did not provide in-depth reporting of qualitative factors affecting implementation. We excluded studies where mHealth was part of a larger complex intervention, studies from high-income countries, and studies that combined analysis of determinants across multiple countries where it was not possible to separate out findings from LMICs versus high-income countries.

### Data Extraction and Analysis

#### Overview

The full search terms and strategy for the databases are detailed in Multimedia Appendix 1. Briefly, we included terms pertaining to LMICs, infectious diseases (eg, communicable disease), and mHealth terms (eg, mHealth, text message, mobile app). We did not include additional filters for qualitative versus quantitative studies.

#### Extracted Data

One reviewer (JGO) screened the titles and abstracts of the search output for relevant studies. As a next step, we conducted full-text screening. Where eligibility criteria were unclear, final consensus on article eligibility was based on discussions with another member of the author team (HMY).

We extracted characteristics of the mHealth intervention including intervention setting (country, LMIC status, health care setting); intervention design; content and purpose; target disease and population; and its quantitative impact on health outcomes originally targeted by the intervention, as reported in the included qualitative study. This was to aid our interpretation of how the qualitative implementation determinants we identified may have affected targeted health outcomes. The original quantitative impact evaluations were not sourced for this review. We also extracted data on details of the qualitative implementation study setting, study population, research question, data collection method, and study size, as well as broad implementation determinants considered by the authors.

#### Data Analysis and Reporting: Tailored Implementation for Chronic Diseases Framework

We performed a framework analysis of determinants affecting implementation based on the Tailored Implementation for Chronic Diseases (TICD) framework [25]. The TICD framework is a comprehensive checklist of determinants of clinical practice developed to inform implementation research projects that are tailored to local conditions [25,26]. The framework can be applied beyond contexts of chronic diseases, as the framework broadly focuses on health system components that determine quality of care. It identifies 7 key domains: guideline factors (clinical care guidelines or mHealth as a “guideline”); health worker factors; patient factors; professional interactions; incentives and resources; capacity for organizational change; and social, political, and legal factors. Its strength lies in its emphasis on ensuring tailoring to local conditions, which is valuable to consider for LMIC interventions, and inclusion of contextual (including political and legal) and patient factors affecting implementation. The TICD framework is a comprehensive health systems framework aligned with the systems thinking framework, which considers interactions with the broader context and patient needs in addition to the structural components of a health system [27].

The framework was used to initially categorize the reported determinants of mHealth intervention implementation and was used to structure the reported findings in the results. Additional emergent themes, such as intervention design and forgetfulness, were extracted.

## Results

### Screening Results

**Figure 1.**
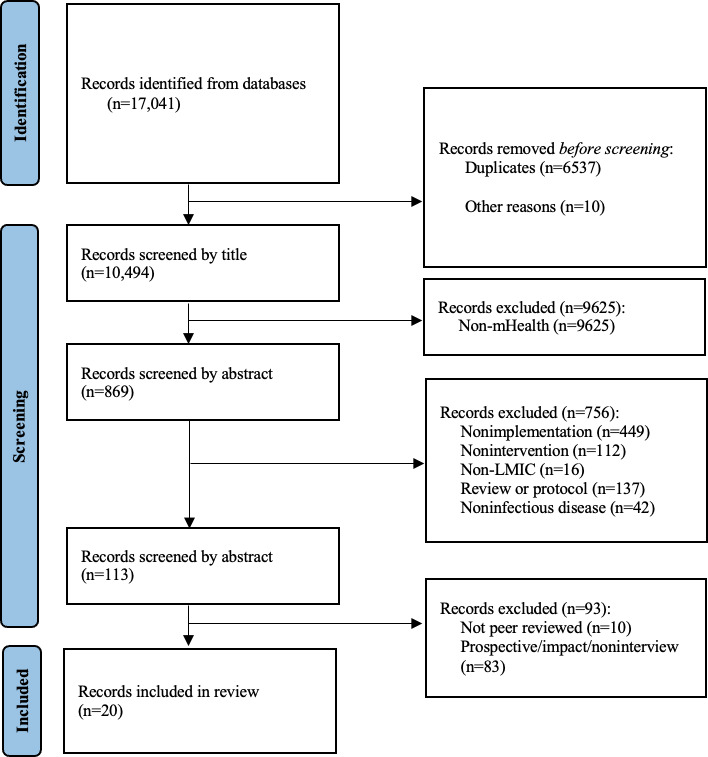
PRISMA flow diagram. Screening strategy and PRISMA reported according to flow diagram for systematic reviews, including database searches. mHealth: mobile health; LMIC: low- and middle-income countries; PRISMA: Preferred Reporting Items for Systematic Reviews and Meta-Analyses.

A total of 17,041 records were initially identified. After removing 6537 duplicates, 10 non-English studies, and 9625 non–mHealth-related studies, a total of 869 studies underwent abstract screening in 2 rounds, each examining different criteria, as seen in Figure 1. A total of 20 studies were included for data extraction and final analysis.

### Characteristics of Included Studies

Details of the intervention including study setting; intervention type and purpose; target disease and population; and quantitative impact evaluations (as reported in the included qualitative manuscripts) are presented in Table 1. Table 2 details qualitative studies analyzed in this review. Among the 20 included studies, 9 were apps and 11 were SMS interventions. Based on the World Bank 2022 Country Group by Income LMIC status [28], 8 were low income [8,9,29-34], 6 were lower middle income [10,35-39], and 6 were upper middle income [7,21,40-43]. Of the 20 studies, 7 focused on people living with or at risk of acquiring HIV [7,21,31,33,34,39,41]; the remaining studies targeted malaria [8,30,35,36], tuberculosis [31,38,42,43], pneumonia [10,30], dengue [37], a grouping of “respiratory illnesses” [9] or “childhood chronic infectious diseases” [29], HPV [40], or COVID-19 [32]. There were 2 studies that addressed multiple diseases [30,31]. Half (10/20) of the interventions targeted health workers [9,10,21,30,31,35,36,39,40,42,43], while the remainder were for patients, individuals at risk of disease, or general community members [7,8,21,29,32-34,37,38,41]. Most studies (11/20) involved community level health care [7,8,10,21,29,32,33,36,37,39,41].

Determinants of mHealth implementation are reported according to the 7 TICD domains and additional emergent themes.

**Table 1.** Characteristics of mHealth intervention among included studies.^a^

Country, author, year, reference	World Bank country income classification	Level of health care; health care context	mHealth intervention	Study design of original quantitative study/comparator	Content of the intervention	Purpose of the intervention	Target disease(s)	Target user(s)	Quantitative impact of mHealth intervention on targeted health outcome(s) as reported in the qualitative study
Argentina, Straw et al, 2023 [40]	Upper middle	Primary; primary hospital and primary health care centers consisting of community health workers	SMS	C-RCT^b^/standard of care	Reminder messages to women with HPV^c^, and one to community health workers about women with no triage 60 days after positive HPV test	Improve Pap triage of HPV-positive women	HPV	HPV-positive women and health workers	Both acceptability of the intervention by HPV-tested women and its adoption by health workers were high: 15% increase in percentage of women with triage Pap after HPV result. Statistical significance not reported
Ghana, Ginsburg et al, 2016 [10]	Lower middle	Community; health centers and community-based health planning and service centers in rural Ghana	App (phone or tablet)	Not reported	Software-based breath counter and a pulse oximeter to count child’s breaths, off-the-shelf reusable pediatric pulse oximeter to detect hypoxia	Improve pneumonia diagnosis and treatment and childhood illnesses in general in accordance with the Integrated Management of Childhood Illness guidelines	Pneumonia	Health workers	Not reported
Kenya, Jones et al, 2012 [35]	Lower middle	Primary; government dispensaries and health centers	SMS	C-RCT/control group	Pediatric outpatient malaria case management accompanied by “motivating” quotes to health worker’s personal mobile phones. Two messages per day across five working days for the duration of the study (26 weeks)	Improve health worker’s malaria case-management practices, specifically drug dispensing and management	Malaria	Health workers	Intention-to-treat analysis showed 24% improvement compared to baseline in correct antimalarial drug management immediately after the intervention, sustained effect of 25% six months later. Statistical significance not reported
Malawi, Ide et al, 2019 [29]	Lower	Community; village clinic in Northern Malawi	App	Not reported	App-directed assessment and management of the visit; the visit was documented in both the app and the village clinic register	Improve assessment, classification, and treatment of seriously ill children, facilitate disease monitoring and surveillance [44]	Childhood infectious disease outbreaks	Health surveillance assistants, caregivers	Not reported
Malawi, Kaunda-Khangamwa et al, 2018 [30]	Lower	Primary and tertiary; health facilities operated by government or private care (including tertiary hospitals)	SMS	C-RCT/control group with no messages	Twice-daily text message reminders on case management of malaria, pneumonia, and diarrhea sent to clinicians and drug dispensers	Improve case management of malaria, diarrhea, and pneumonia	Malaria, diarrhea, pneumonia	Health workers	Nonsignificant, 4% improvement in correct malaria case management. Statistical significance not reported
Mali, Mangam et al, 2016 [8]	Lower	Community; rural district community	SMS	3 pilot intervention villages against 3 nonintervention villages	Educate and instruct households about indoor residential spraying campaign	Lower malaria prevalence through preventative measures	Malaria	Households	Significantly lower among the mobile-messaging villages than the door-to-door mobilization villages (86% vs 96%, respectively; *P*=.02) and significantly lower structural preparedness in households mobilized through the mobile-messaging approach compared with the door-to-door approach (household and food items removed; 49% vs 75%, respectively; *P*=.03)
Mozambique, Nhavoto et al, 2017 [31]	Lower	Primary; health care centers providing intense ART^d^ and tuberculosis care	SMS	RCT^e^/no comparator	Structured series of SMS text messages sent automatically based on appointments and scheduled drug pickups. Messages were sent 7 and 2 days before appointment or drug pickup	Support retention in ART and tuberculosis treatment	HIV and tuberculosis	Patients and health workers	The majority of HIV patients (61/68, 90%) and the majority of tuberculosis patients (60/68, 88%) reported not having missed any appointments.Majority of the patients (HIV: 56/68, 82%; tuberculosis: 65/67, 97%) reported not having missed medication pickup at any time
Myanmar, Win Han et al, 2021 [36]	Lower middle	Community; ICMV^f^ managed by Myanmar’s National Malaria Control Programme and its implementing partners	App (phone)	Mixed method/paper-based reporting control group	Malaria case-based data entered by ICMVs directly in the app on their mobile phones, which is instantly uploaded onto the dedicated District Health Information System 2 database	Enabled more accurate and complete data reported to improve integrated community malaria volunteers’ malaria prevention, diagnosis, treatment and referral services	Malaria	Health workers	Not reported
Nepal, Bhattarai et al, 2019 [37]	Lower middle	Community; rural households in district highest in dengue outbreaks	SMS	Nonrandomized quasi-experimental design. Control group (standard of care), dengue prevention leaflet only group, dengue prevention leaflet with mobile SMS intervention group	Disseminate preventative health messages via mobile phone twice per week	Change in dengue preventative knowledge and practice of respondents	Dengue	Households	Dengue prevention leaflet and SMS intervention combination significantly improved dengue prevention knowledge (mean 32.7, SD 13.7 vs mean 13.3, SD 8.8) and practice (mean 27.9, SD 11.4 vs mean 4.9, SD 5.4) compared to without SMS (*P*<.001)
Pakistan, Mohammed et al, 2012 [38]	Lower middle	Secondary; hospital in low-income industrial area (free)	SMS	No control group	Daily reminders sent and patients were asked to respond after taking their medication. Motivational message, followed by a reminder to respond to the system	Improve tuberculosis drug adherence	Tuberculosis	Patients with tuberculosis	Mean response rate of 57% for all participants.The mean response rate fell from 62% during the first 10 days to 49% during the last 10 days across the 30-day intervention. Statistical significance not reported.
Russian Federation (Siberia), Hodges et al, 2022 [7]	Upper middle	Community; inpatient and outpatient settings in the tuberculosis referral hospital and AIDS center	App (phone)	Not reported	Daily patient check-ins or queries regarding stress, mood, and ART adherence; appointment reminders; tailored educational resources; access to HIV-related lab results; community message board for anonymous peer messaging; direct messaging with clinic care team members outside of the clinic	Enhance linkage of HIV/tuberculosis coinfected patients to HIV care and promote sustained engagement with and integration of HIV and tuberculosis care	HIV	People with HIV	Improved rates of linkage to care at the AIDS center, improved medication refill rates, reduced virologic failure at 6 months on ART. Related statistics were not reported.
Rwanda, Babili et al, 2023 [32]	Lower	Community; home-based care	SMS	Not reported	Automated check-in message sent daily for 14 days, contact tracing, and data centralization	Real-time remote monitoring and support of COVID-19 cases and contacts	COVID-19	Patients with COVID-19	Nonresponse rate 25%‐30%
South Africa, Adeagbo et al, 2021 [41]	Upper middle	Community; rural and periurban HIV hyperendemic area	App (tablet)	C-RCT/standard of care	Enable user to hear the “story” of a chosen character portrayed on the app	Improve HIV testing and linking with care in rural South Africa	HIV	Male participants at risk of HIV	83% consented to and used a home-based rapid HIV test, 33% received HIV testing for the first time in the annual HIV surveillance. Statistical significance not reported.
South Africa, Chaiyachati et al, 2013 [42]	Upper middle	Tertiary; decentralized MDR-TB^g^ treatment centers in rural South Africa, part of larger MDR-TB hospital	App (phone)	Not reported	Reporting of adverse effects of MDR-TB, decision aids for triaging symptoms complaints, adherence questions, and a tool for tracing newly diagnosed tuberculosis patients or finding defaulters from tuberculosis treatment	Improving the acceptability feasibility of clinical monitoring and management of adverse events in patients receiving community-based MDR-TB treatment	MDR-TB	Health workers	Low user uptake: 27% of health workers submitted adverse event forms through the mHealth app
South Africa, Janssen et al, 2020 [21]	Upper middle	Community; community township–based HIV clinics [45]	App	Not reported	Video that shows the user how to conduct the oral self-test, guide on how to interpret test results, information about HIV and HIV transmission, HIV risk assessment with questions regarding a person’s sexual behaviors, condom use, and alcohol and drug use	Support HIV self-testing and care	HIV	People presenting to HIV self-test clinics	Not reported
South Africa, Maraba et al, 2018 [43]	Upper middle	Primary; primary health clinics	App and SMS	Quantitative comparisons between preintervention and postintervention	Data collection, automate results delivery, display results, provide notifications, and directly provide results via results notification and via text	Reduce the time and effort required for tuberculosis data reporting, provide rapid and automatic access to Xpert MTB/RIF tuberculosis test results	Tuberculosis	Health workers	No statistically significant difference in results between paper-based system and mHealth-based system in terms of details documented, proportion on tuberculosis treatment, and time before results availability
Uganda, Ellington et al, 2021 [9]	Lower	Primary; primary health care facilities, one periurban and one rural	App	Not reported	Decision support tool, partially automated respiratory rate counter, educational videos, adapted respiratory assessment score to determine bronchodilator responsiveness	To improve diagnosis and treatment of acute lower respiratory infections in children <5 years of age	Acute lower respiratory illness	Health workers	Not reported
Uganda, Haberer et al, 2010 [33]	Lower	Community; outpatient HIV clinic at regional rural referral hospital	SMS and interactive voice recording	Not reported	Users were expected to respond to a question regarding HIV ART treatment adherence sent via SMS, unlocked via personal identification number prompt	Automated collection of weekly individual-level ART adherence data	HIV	Caregivers of HIV-positive patients	76% of the SMS cycles were not answered, meaning that no response was received to the greeting and prompt for the personal identification number necessary to respond to the adherence question
Uganda, Twimukye et al, 2021 [34]	Lower	Primary; HIV clinics from a periurban government health facility	SMS and interactive voice recording	RCT/standard of care	Interactive voice recording calls or SMS text message appointment reminders on or before the scheduled appointment date. Allows patients to report symptoms at the end of the scheduled call through a toll-free line	Promote adherence to ART for young adults	HIV	HIV-positive youth	Not reported
Zimbabwe, Venables et al, 2019 [39]	Lower middle	Community; health centers in rural settings	SMS	Not reported	HIV viral load testing results sent to health facilities or patient	Reduce the amount of waiting time for test results and improve adherence support	HIV	Health workers and patients	Median waiting time from reporting of the viral load result at the laboratory to starting enhanced adherence counseling was reduced from 47 days to 30 days compared to preintervention

a mHealth: mobile health.

b C-RCT: clustered randomized controlled trial.

c HPV: human papillomavirus.

d ART: antiretroviral therapy.

e RCT: randomized controlled trial.

f ICMV: integrated community malaria volunteers.

g MDR-TB: multidrug resistant tuberculosis.

**Table 2.** Characteristics of qualitative study design among included studies.

Country, author, year, reference	Study population	Research question	Data collection method (study size)	Reported qualitative factors affecting user uptake
Argentina, Straw et al, 2023 [40]	Health decision makers, health workers	Stakeholder’s perception about mHealth^a^ implementation strategy and factors affecting scale-up	Semistructured interviews (n=20)	Knowledge of the strategy Characteristics of the intervention (intervention source; design quality and access to knowledge and information; adaptability; complexity; and compatibility with norms, values, and existing workflows and systems; relative advantage; consideration of patient needs; relative priority; leadership engagement; external policies; cost)
Ghana, Ginsburg et al, 2016 [10]	Health administrators, health workers, caregivers	Feasibility, usability, and acceptability of the app in 6 health centers and 5 community-based health planning and services centers	In-depth interviews (n=69)	Feasibility of the app: national-level support, integration into routine childhood care, electricity needs Usability of the app: easy-to-use design, improvement over manual assessments, time constraints with full assessment Acceptability, algorithm perception as accurate
Kenya, Jones et al, 2012 [35]	Health workers	Perceptions and experiences of health workers involved in the mHealth intervention	Interview (n=24)	Perception of the intervention based on app design Clinical importance of practice Relationship to training, guidelines, and other interventions
Malawi, Ide et al, 2019 [29]	Health surveillance assistants, caregivers	Acceptability and impact of the app	Semistructured interviews (n=40)	Health surveillance assistant and caregiver acceptability and beliefs: evidence strength and quality, tension for change, beliefs about the app Technical and clinical characteristics of the app: impact on clinical assessments, learning curve, features of the app, relative advantage of the app over standard care Technological infrastructure Caregiver-health surveillance assistant relations
Malawi, Kaunda-Khangamwa et al, 2018 [30]	Drug dispensers and health workers	Health worker perceptions of the messages received, possible mechanisms of action, and potential challenges to acting on the SMS reminders with the overarching goal of understanding the reasons why the intervention was ineffective and elucidating lessons learned	Semistructured interviews (n=50)	Perceptions of the SMS intervention Design of SMS intervention Health facility resources: staff, finance Communication between health workers
Mali, Mangam et al, 2016 [8]	Pilot village community members	Pilot effectiveness was investigated by evaluating structure preparedness	Interview (n=18), survey interview (n=673)	Language literacy (writing and reading) Perceptions and attitudes about the intervention due to familiarity with mobile phones Enumeration
Mozambique, Nhavoto et al, 2017 [31]	Patients, health workers	Patient and health worker views on an mHealth intervention aiming to support retention in ART^b^ and tuberculosis treatment in Mozambique	Semistructured interview (n=181)	Perceptions of participants toward the SMS system
Myanmar, Win Han et al, 2021 [36]	Community malaria volunteers, malaria program stakeholders	Qualitative assessment of the sustainability prospects of the reporting system in the context of Myanmar’s malaria elimination program	FGDs^c^ (n=84); semistructured, in-depth interviews (n=14)	User satisfaction, system access Ownership, human resources, financial sustainability, system applicability (net benefits), policies and operation procedures Technological system interoperability, system scalability, system relevance, system quality
Nepal, Bhattarai et al, 2019 [37]	All household heads or spouses, SMS recipients, key informants from stakeholder organizations (dengue focal points, public health officer)	Effectiveness, acceptability, and appropriateness of the mobile SMS intervention in improving behavior in dengue endemic areas of Nepal	In-depth interviews (n=13), survey interviews (n=300)	Network Mobile phone familiarity, existing practices Financial cost Organizational and social responsibility Organizational readiness Intervention design: entertainment value, informative nature, timing of messages Perceived usefulness
Pakistan, Mohammed et al, 2012 [38]	Patients	Perceptions, acceptability, and engagement with an interactive SMS reminder system for patients with tuberculosis	Semistructured interviews (n=24)	Literacy Ownership of a mobile phone and familiarity with SMS messaging Familiarity with tuberculosis treatment adherence Technological problems
Russian Federation (Siberia), Hodges et al, 2022 [7]	People with HIV treated at the AIDS center, people with HIV and tuberculosis treated at the referral hospital, clinical and nonclinical providers from the tuberculosis hospital and AIDS center	Process evaluation for adaptation, testing, and dissemination of the mHealth program	Unstructured group interviews (n=30), community message board sampling survey (n=47)	Language adaptation of platform components Server management and technological resources Iteration of platform features Program organizational practice integration Communication about the intervention
Rwanda, Babili et al, 2023 [32]	Senior staff (policymakers, directors, and senior managers), technical teams (case managers and health workers supporting intervention implementation)	Rationale, perspectives, and experiences of stakeholders during mHealth intervention implementation and the intervention’s scalability and adoptability	Semistructured 1-on-1 interviews (n=7)	Local governance and policies Local infrastructure Intervention characteristics End user characteristics Local culture and communication
South Africa, Adeagbo et al, 2021 [41]	Men aged >15 years	Acceptability of and satisfaction with the intervention	In-depth interviews (n=20), qualitative survey interviews (n=232)	Relevance and convenience Familiarity of practice based on existing behavior Ease of use
South Africa, Chaiyachati et al, 2013 [42]	Mobile health workers	Evaluate acceptability and feasibility of using the app to record and submit adverse event forms weekly; evaluate mobile health workers’ perceptions throughout the pilot period	2 in-depth FGDs with health workers (group size n=5)	Forgetfulness Technological functionality Responsibility of care Ease of use
South Africa, Janssen et al, 2020 [21]	Nurses and health workers, patients	Affective dimensions of HIV self-testing using a smartphone app strategy	Interview (n=30), 1 FGD (group size n=6)	Past interactions with health care system or experiences with HIV testing Perception of the app
South Africa, Maraba et al, 2018 [43]	Tuberculosis patients, health workers	Feasibility, acceptability, and potential of an mHealth app to reduce initial loss to tuberculosis follow-up	Structured interviews (n=29)	Perceived benefit (patients and health workers) Technical difficulties Proficiency in receiving text messages/understanding the intervention
Uganda, Ellington et al, 2021 [9]	Health facility administrators, primary health workers (nurses, clinical officers)	Health workers’ perceptions of acceptability, usability, and feasibility of the app	Semistructured interviews (n=3), 3 FGDs (n=25)	Acceptability and perceived benefit of the app, app usability, provider-patient relationship Health facility resources, eg, human resources, supply chain App integration into existing health system Stakeholders buy-in
Uganda, Haberer et al, 2010 [33]	Caregivers, intervention participants	Participant impressions of the technologies	Qualitative interview (n=19)	Previous experience with mobile phones Understanding how to respond to the interactive voice recording and SMS prompts Network access and technological resources
Uganda, Twimukye et al, 2021 [34]	Young adults (aged 18‐25 years) with HIV infection	Acceptability and feasibility of a mobile phone support tool to promote adherence to ART among young adults in a randomized controlled trial	In-depth interview (n=11), 1 FGD (group participants n=21).	Perceptions toward app Technical issues, access to mobile phones Stigma
Zimbabwe, Venables et al, 2019 [39]	Patients, health workers	Patient and health care worker experiences and perceptions of the SMS intervention	In-depth interview (n=32), FGD (n=5)	Patients’ understanding of messages Perceived benefits of text messages Organizational personnel leading the intervention Technology resources

a mHealth: mobile health.

b ART: antiretroviral therapy.

c FGD: focus group discussion.

### Determinants of mHealth Implementation: TICD Framework

#### Guideline Factors

There were 6 studies that reported on how lack of clarity and understanding of the intervention hindered its uptake. For example, users did not respond to mHealth intervention text message prompts because they were uncertain about how to use the personal identification number, were uncertain about how to respond to the message, or received little to no information about the background of the intervention [7,30,33,39,40,43]. Compatibility of the intervention with users’ past experiences with either the technology and its use in health care, or the intended change in health care practice, was a key facilitator that aided in implementation. Existing familiarity with the intended behavior promoted by the intervention—such as antiretroviral treatment adherence, HIV testing, or general interactions with the health care system—facilitated uptake [21,29,35,38,41]. The mHealth intervention in Babili et al’s study—WelTel, assessed for COVID-19 case and contact management—was previously implemented for HIV epidemic virtual care, which facilitated its implementation as users were familiar with the platform and its functionality [32]. Similarly, health workers commented on how an app’s alignment with existing practices of using the village clinic register increased the likelihood of engagement [29]. Existing mobile phone use or interest in the use of new technologies were additional facilitators, as users were already familiar with making appointments, making calls, or using SMS technology [8-10,32,33,36,37,42]. Trustworthiness of the source of the recommendation given by the mHealth intervention facilitated implementation, particularly when the mHealth intervention intended to aid health workers in improving their health care practice or disease management [10,29,32,35,40].

#### Individual Health Care Worker Professional Factors

mHealth being perceived as useful by end users in improving existing health care practices facilitated the uptake and integration of the intervention. For instance, after initial use of the intervention, a perception that it might improve existing clinical practices, improve patient engagement with services, or relieve strain on the health system were key for implementation [9,29-32,35,36,39,40,43]. Health workers in Ide et al’s study perceived the app as advantageous over existing systems, as the intervention aided in conducting more accurate, error-free community case management of malaria, diarrhea, and pneumonia, which facilitated uptake [29].

Furthermore, for interventions for which the main users were health workers, attitudes toward the intervention were impacted by the perceived effect of the use of the intervention on the health worker’s reputation. Ellington et al identified that the perceived loss of trust between patients and health workers in the health worker’s ability to diagnose and treat patients due to their reliance on technology to deliver health care was a barrier to using the app [9]. In contrast, Ide et al commented on how the app facilitated perceived professionalism [29]. Twimukye et al commented on how the health worker’s use of the app improved how the patient perceived the health worker’s attention to detail and care [34].

#### Patient Factors

Patients perceiving the intervention as beneficial in improving health outcomes through increased convenience, awareness, or reminders facilitated implementation of the intervention [34,38,41,43]. Adeagbo et al commented on how the app’s positive messages about HIV testing and adherence promoted users to adopt new behaviors by improving individual competency to make informed, healthy decisions concerning sexual health [41]. Language literacy was a barrier that limited engagement with 2 SMS intervention studies targeted at patients or community members [8,38].

#### Professional Interactions

Limited supervision and follow-up of the intervention implementation by the research team leading the intervention was noted as a barrier, as users requested feedback and confirmation of correct intervention use [30]. Lack of or limited access to training to provide necessary skills to effectively engage with the mHealth intervention was a barrier to implementation for health workers [9,36]. Access to professional training was a facilitator of implementation [10,29,40].

#### Incentives and Resources

Several studies reported that access to resources and essential infrastructure influenced implementation. Specifically, 6 studies reported that poor telecommunications networks, problems with electricity, a lack of phone coverage, limited staffing, and a lack of equipment to implement the behavior change were barriers to implementation [10,30,33,37,40,42]. Network problems were particularly prominent in geographically remote areas. Other barriers included technology that repeatedly malfunctioned (such as periodic freezing and system crashes) and limited access to technology support systems to troubleshoot technological problems [7,29,32-34,36,38,42,43]. Access to technological resources and support in case of malfunctions facilitated implementation [33].

#### Capacity for Organizational Change

Financial instability, existing patient overflow, incompatible technological equipment, and length of appointment time within the clinic hindered the implementation of mHealth interventions [7,9,30,37,39]. Kaunda-Khangamwa et al reported that 90% of the health worker respondents blamed existing high workloads and drug stockouts as factors discouraging health workers to respond to SMS reminders that promoted infectious disease case management [30]. Similarly, Ellington et al’s study identified that the existing length of the appointment time was not compatible with mHealth intervention use as the time to complete a health assessment through the app took longer than the appointment duration [9]. Straw et al commented on the compatibility of the mHealth intervention with the existing organization functionality to facilitate normal workflow as a facilitator of implementation [40]. A lack of management and ownership of the intervention by health workers further contributed to a limited capacity for embedding the intervention within the health care facility [39].

#### Social, Political, and Legal Factors

One study commented that the costly nature of airing messages during the daytime and limited funding for the intervention were barriers to successful implementation [42]. Receiving national-level support on a political level including the Minister of Health or district leadership was a facilitator of implementation [9,10,32]. Babili et al commented on how the Rwandan government’s support of digitization across all governmental sectors by offering resources for implementation aided the adoption of the digital health intervention [32]. Furthermore, shared responsibility and corporate social responsibility felt among the wider community was an enabler, as the charitable community assisted in reducing costs and improving uptake [37].

### Emergent Themes

A novel factor not directly addressed in the TICD framework is the importance of considering app or SMS design features. Frequency of messages, language, and integration of local narratives to engage users were reported as affecting ease of use and user uptake [9,10,30,34,35,37,41,42]. mHealth interventions that adapted content toward the intervention context by using local proverbs, narratives, or language facilitated uptake of the intervention [7,30,35,41]. Moreover, features such as a user-friendly interface and a streamlined workflow facilitated implementation, while an intervention design that increased the workload of health workers was a barrier to implementation [32].

The study by Mangam et al, whose reminder SMS system replaced the existing door-to-door reminders, uniquely commented on how the absence of face-to-face reminders increased the rate of forgetfulness and patients ignoring the messaging, thereby affecting its implementation [8].

### mHealth Impacts on Target Health Outcomes and How Implementation Determinants May Have Influenced These Outcomes

As described in Table 1, there were 8 studies that identified that mHealth had a positive impact on health outcomes or behavior, whether that was through increased HIV testing, reduced errors in drug and disease management, or improved disease prevention knowledge, results collection, or linkage to care for better clinical practice [7,29,31,35,37,39-41]. In 4 studies, the mHealth intervention did not lead to an improvement in the health outcome—responses to SMS prompts were low, preventative measures were worse than in the non-mHealth control group, or user uptake was low [8,33,38,42]. There were nonsignificant changes in health outcomes or behavior in 2 studies [30,43]. Quantitative health outcomes were not reported in 6 of the reviewed studies [9,10,21,32,34,36].

Improved outcomes may be explained by familiarity with the health behavior or technology [7,29,35,37,41], positive attitudes among health workers toward the technology [29,31,35], or ease of use of the mHealth technology [7,35,37,41]. Technological barriers, lack of familiarity with technology, and resource limitations [8,30,33,38,42,43] may have reduced engagement with the intervention or the participants’ ability to implement the behaviors enforced by the intervention, therefore diluting the intervention effect.

## Discussion

### Principal Findings

**Figure 2.**
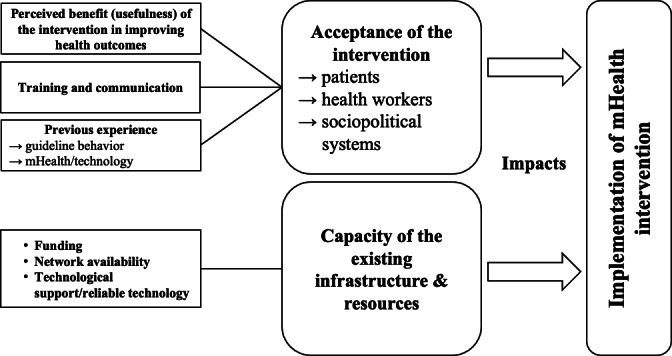
Model of factors influencing implementation of mHealth interventions in LMICs. The two main factors are presented in large boxes: (1) acceptance of the intervention and (2) capacity of the existing infrastructure and resources to accommodate mHealth. Acceptance, in turn, was mainly influenced by perceived usefulness of the intervention, amount of training and communication, and previous experience with the guideline behavior and mHealth or technology. The health care system’s capacity and infrastructure and resources were influenced by funding, network availability, and technological support. mHealth: mobile health.

**Figure 3.**
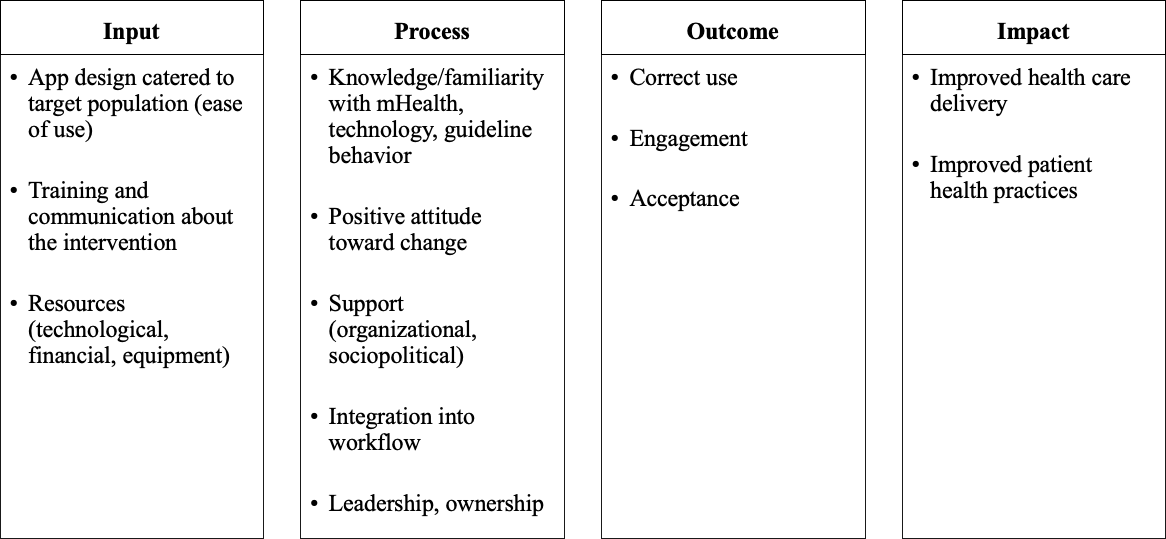
Logic diagram depicting determinants of successful mHealth implementation. Overview of mHealth implementation determinants from the mHealth design and resource input stage to the process of implementation and the desired outcomes and impact. mHealth: mobile health.

Findings from the 20 reviewed studies and categorization into the TICD framework were synthesized to deduce two overarching themes that influenced the successful implementation of mHealth initiatives in LMICs: (1) the acceptance of the intervention by patients and health workers (as well as on a sociopolitical level), regardless of the target user, and (2) the capacity of existing infrastructure and resources to implement the intervention, which was strongly tied to the health system’s capacity for change. This relationship is visually depicted in Figure 2. The logic flow diagram in Figure 3 further represents these reported factors according to inputs required for mHealth interventions and the required processes for success.

### Acceptance of the Intervention

#### Overview

Acceptance of the intervention was strongly linked to uptake of the intervention, which facilitated implementation of the intervention and its potential to improve health outcomes. Patients and health workers were influenced by the perceived benefits of the intervention in improving health outcomes, the extent of training, and previous experience with the guideline behavior or mHealth.

#### Perceived Benefit of the Intervention

Perceived benefit of the intervention was influenced by ease of use and design of the intervention [9,10,30,32,34,35,37,41,42]. Where an app or SMS design engaged users in a way that improved health outcomes, patient engagement with health care services, or health care efficiency, participants were less likely to perceive the use of the mHealth technology as burdensome, facilitating successful implementation [9,29-32,34-36,38-41,43]. An intervention that provided a clear advantage compared to existing practice facilitated the perception of it being beneficial, as supported by the intervention’s design and ease of use. These findings were consistent with Davis’s Technology Acceptance Model and its application in health care, which suggests that perceived ease of use and usefulness both affect attitudes toward and use of technology [46,47]. Design considerations was an emergent factor unique to mHealth and technology-related interventions, emphasizing the value of iterative design of the mHealth intervention before implementation [9,10,30,34,35,37,41,42]. Further reviews reporting on mHealth in the context of antiretroviral therapy adherence and maternal health have identified the importance of intervention design, including tailoring SMS messages and the frequency of reminders [48,49].

Political support of the intervention ensured effective implementation; this included stakeholder buy-in and approval of the intervention, and support from health administrators on a district level [9,10,32]. The importance of understanding the need for the intervention to reduce disease burdens or improve health care services is supported by a previous review by Opoku, Stephani, and Quentin [50]. Therefore, regardless of the target user, perceived benefit of the intervention is crucial for implementation of the initiative, emphasizing the community-wide, integrated nature of mHealth interventions.

However, studies by Kaunda-Khangamwa et al and Mangam et al noted that, despite the users’ positive attitudes toward the intervention, factors such as lack of communication regarding intervention use and limited resources to implement the behavioral guideline hindered implementation [8,30]. Perceived value of the intervention and acceptance alone therefore cannot guarantee successful implementation and positive outcomes from an intervention.

#### Training and Communication

Users who received communication and training on the intervention and its use before or during its implementation engaged well with the intervention and intended guideline practice [9,10,29,36]. Lack of awareness and clarity regarding how to engage with the intervention were consistently noted across studies with limited user engagement [30,33,43]. Sufficient training has been previously noted in a review as a contributing factor to mHealth intervention implementation, which is closely linked to the perceived ease of use [2]. For interventions aimed toward health workers, training was either provided by the intervention research team members or between health workers; follow-up on correct use was identified as being important for encouragement and continued intervention use [7,9,10,30,42].

#### Compatibility With Existing Health Care Practices and Social Norms

##### Familiarity With the Guideline Recommendation

Among studies that had high engagement with mHealth and improved health outcomes, existing familiarity with the guideline behavior, such as treatment adherence or HIV testing, was recurringly noted as a facilitator of implementation [7,37,39,41]. This suggests that successful mHealth initiatives complemented normative behavior, existing health care practices, and “new” practice (eg, interventions to improve treatment adherence), given their importance was already understood. This suggests mHealth has limited value in establishing new behavioral practices but rather is advantageous in complementing existing practices. Compatibility with existing behavior was strongly linked to the perceived benefit of the intervention; when the intended guideline was not yet an established practice or initially perceived as useful, mHealth interventions that encouraged this behavior were less likely to be accepted by the user [21,29,35,38,41]. Ide et al’s study commented on how the mHealth intervention facilitated existing practices of childhood infectious disease management and improved reliability of the tests [29].

Although social norms (such as stigma) were only reported in 1 study [34], a number of studies commented on how users who were already comfortable sharing diagnoses or their health status were more engaged in the mHealth intervention, suggesting mHealth success is dependent on existing social norms and behaviors [34,39,43].

##### Attitudes and Familiarity With mHealth

Lack of familiarity with mobile phone use was a clear barrier to implementation; this barrier was particularly noted in studies with limited successes [8,43]. An existing understanding of the benefit of mHealth interventions—or previous positive experiences with mHealth—also affected user uptake. This was indirectly seen in mHealth intervention uptake being influenced by the perceived impact of use on provider-patient relationships [9,29,34]. This factor was conflicting across multiple studies, as Ide et al and Twimkukye et al commented on how the mHealth intervention facilitated perceived professionalism and improved provider-patient relationships, while Ellington et al noted the perceived decreased patient trust of the health worker to diagnose and treat the patient [9,29,34]. A systematic review of maternal health interventions also identified that technological literacy and previous experience of mHealth use were enablers of mHealth uptake, among a range of other factors [51,52].

### Capacity of Existing Infrastructure and Resource Availability

The importance of capable infrastructure and resource availability have been noted by existing systematic reviews as key determinants of implementation [2,5,15,50-52]. Reviewed studies further confirmed this and noted the importance of staffing, network availability, technological support, and reliable technology in facilitating the implementation of the intervention [9,29,33,34,36-38,42,43]. These factors were tightly linked to social, political, and legal factors (such as limited staff funding or unstable network coverage to remote areas); these barriers often reflected the greater health care system’s resourcefulness. Analysis of the factors influencing implementation therefore emphasized the interaction of health system components and the importance of considering the broader context beyond the health care system, as described in the systems thinking framework [27]. It is possible that due to the targeted, narrow nature of some of interventions, there was insufficient technological support or insufficient resources for successful implementation. Increased health data reporting on a health care system level could also contribute to improved resource allocation and policy decisions from sociopolitical organizations that could aid in mHealth implementation [53]. This further emphasizes the importance of understanding the value of the intervention in improving health outcomes across all stakeholders, as it could result in securing increased funding for the improved implementation of the initiative.

Further determinants of implementation included the system’s capacity for change, such as how the intervention fit into existing appointment durations and organizational leadership structures [9,36]. This limited capacity for change could reflect the unstable foundation and support of the health care systems within these communities and indicates a potential lack of preparation for future changes or health challenges. Existing reviews on mHealth implementation have also commented on the importance of considering the existing health care system, such as government funding and capacity, when implementing mHealth interventions [2,15,16].

### Strengths and Limitations

This review is valuable in its consideration of findings across a range of different LMIC settings in Africa and Asia, with a particular focus on periurban and rural areas. The diversity of study settings provides a broad range of factors to consider during implementation in different LMIC contexts. This review synthesized findings by drawing on a comprehensive health systems framework [25] and additional themes, further contributing to its novelty.

However, our review had several limitations. First, most studies (15/20) were conducted in Africa, limiting generalizability to other regions. Second, it was challenging to weigh the relative importance of each implementation determinant in each study, as the included studies were all qualitative. Regardless, the findings provide insights that quantitative results would not have been able to capture. Third, all studies were limited in that data were mainly from patients, health workers, administrators, or assistants, rather than from a sociopolitical level. Fourth, we did not source the primary quantitative impact evaluations of mHealth interventions. For further study, quantitative outcomes from impact studies can be better correlated with the specific determinants—and their respective strength of association—of implementation.

### Recommendations

#### Overview

Insights from this review can help shape health policies and identify key considerations when developing mHealth interventions to improve their efficacy and sustainability in improving health outcomes. A full list of recommendations as reported in each study is noted in Multimedia Appendix 2.

**Figure 4.**
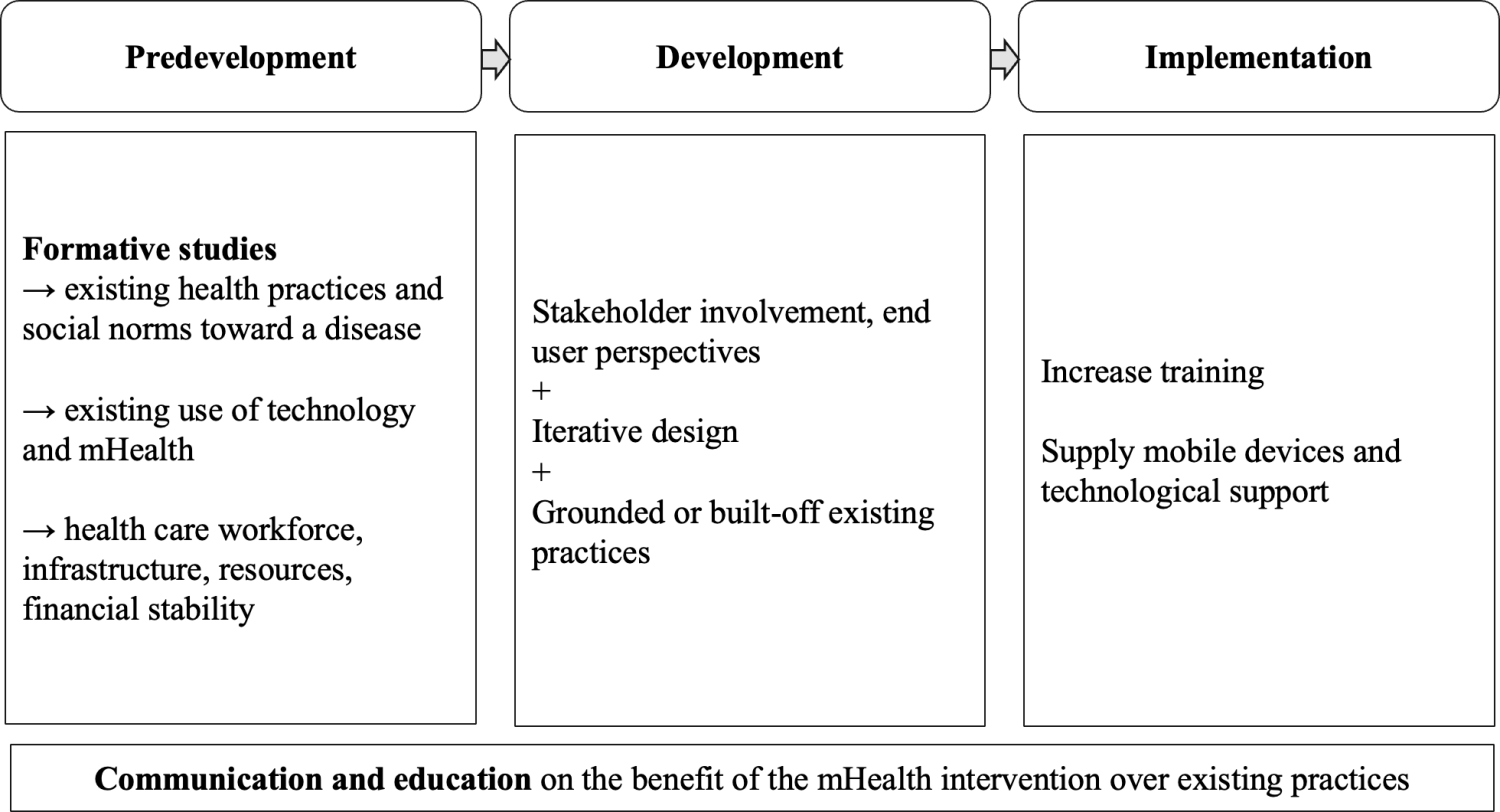
Identified recommendations for successful implementation of mHealth interventions. mHealth: mobile health.

When interpreting findings, it is important to consider the different contexts within which mHealth interventions are implemented, as they must be tailored to the context. Several considerations are important during predevelopmental, developmental, and implementation phases of the mHealth intervention, as seen in Figure 4.

#### Predevelopmental Considerations

A thorough understanding of existing health and technology practices and social norms toward a disease are crucial to predict the feasibility of an mHealth intervention in a specific context. This understanding of the cultural context and structural factors—such as the broader health care workforce, capacity of existing infrastructure, and resource availability—can be achieved through formative research [42]. Communication and education about the importance of health care practices and mHealth’s ability to facilitate health can aid in the acceptance of the mHealth intervention.

#### Development of the Intervention

There were 4 studies that performed prepilot testing and designed the intervention iteratively to maximize participant engagement with the intervention during implementation [7,8,31,42]. For example, through pilot testing, Mangam et al identified the need to incorporate interactive voice messaging in their SMS intervention, as many users were unable to read or understand the texts [8]. To complement the theoretical foundations of an intervention, studies emphasized the importance of stakeholder involvement in the iterative design process during the development of the initiative [2,8,15,16,19,20,42].

#### Implementation of the Intervention

As most interventions require a change in knowledge or behavior, it is important to communicate the benefit of the mHealth intervention compared to existing practice, prior to and during implementation. Increased training will increase confident user engagement with the intervention for long-term improvement of health outcomes [10,33,36]. Supplying mobile devices and offering technological support were recommended by studies to mitigate technological barriers [9,10,36].

It is important to tailor mHealth interventions to complement existing health services and face-to-face practices to optimize the desired health outcome. Adeagbo et al commented on how the mHealth intervention alone was insufficient in completely removing the barrier of accessing HIV testing and health care services [41]. Mangam et al discussed how future mHealth-based mobile communication should complement the community’s existing familiarity with interpersonal communication, particularly as their SMS notifications of health prevention measures were not met with improvement compared to the non-mHealth, face-to-face status quo [8]. An example of complementing mHealth with non–technology-based communication is seen in the study by Bhattarai et al, who paired SMS text messaging with pamphlets [37].

### Conclusion

This review provided comprehensive insight and an analysis of factors influencing the implementation of mHealth initiatives in LMICs. This review underscores the importance of iterative development of the intervention and deep consideration of the structural factors and cultural context before mHealth implementation to ensure scalability and sustainability to improve communicable health outcomes in LMICs.

## Supplementary material

10.2196/55189 Multimedia Appendix 1 Search terms for databases.

10.2196/55189 Multimedia Appendix 2 Reported gaps and recommendations.

## Abbreviations

LMIC: low- and middle-income countries
mHealth: mobile health
PRISMA: Preferred Reporting Items for Systematic Reviews and Meta-Analyses
TICD: Tailored Implementation for Chronic Diseases
WHO: World Health Organization
