# Supplementary material for: Qualitative Evaluation of mHealth Implementation for Infectious Disease Care in Low- and Middle-Income Countries: Narrative Review
Source: JMIR Mhealth Uhealth. 2024 Dec 13;12:e55189. doi: 10.2196/55189 (PMC11660726; doi:10.2196/55189)
Supplement: Multimedia Appendix 2 [file mhealth-v12-e55189-s002.docx]

**Reported Gaps and Recommendations**

| **Study** | **Reported Gaps or Recommendations for future implementation** |
| --- | --- |
| Adeagbo *et al*. 2021 | - App should work alongside other intervention – mHealth intervention is insufficient to overcome barriers to accessing HIV testing and care services |
| Babili *et al.* 2023 | - Improving interoperability across different platforms |
| Bhattarai *et al*. 2019 | - Recommended planned coordination with the private network providers |
| Chaiyachati *et al*. 2013 | Before implementation:   - Understand why health workers may not be using mHealth as expected or desired - Understand the relationship between health workers and patients within the cultural context - Consider the end-user perspective - Consider everyday work constraints in clinicians’ and support staff’s lives   Need to understand the total work environment and the work demand |
| Ellington *et al*. 2021 | - Design-specific recommendations e.g. additional automated or semi-automated smartphone tools, store clinical information, extend to other disease processes and age groups, adjust visual display, layout and app flow - Incorporate children’s risk stratification and environmental risk factors to focus on prevention |
| Ginsburg *et al*. 2016 | - Provide extra devices in the clinic that can be used during power outages or when tablets are out of charge - Caregivers need information and counselling on the app - Pair app with other health care training |
| Haberer *et al*. 2010 | - Improvement in training: hold repeated trainings throughout implementation and use, group training and test knowledge from the training to verify understanding |
| Hodges *et al*. 2022 | Not reported |
| Ide *et al*. 2019 | Not reported |
| Janssen *et al*. 2020 | Not reported |
| Jones *et al*. 2012 | Not reported |
| Kaunda-Kahngamwa *et al*. 2018 | - Need to improve SMS supervision - More financial incentive |
| Mangam *et al.* 2016 | - Add radio announcements and community town hall meetings to supplement the mHealth intervention and ensure the messages reach a wider community. Added human aspect to an approach that lacks traditional social interaction - Use a locally based telecom provider to reduce the overall costs of sending mass messages, possibly an open-source mass text messaging service |
| Maraba *et al*. 2018 | Not reported |
| Mohammed *et al.* 2012 | - For illiterate individuals a 'missed call' system may be helpful, whereby participants call a number but if no one answers the call does not incur a cost for the participant |
| Nhavoto *et al*. 2017 | - Offer free mobile phones to users who cannot afford one, or partner with mobile phone operators who could donate phones, SIM cards - Medical practitioners be able to access patient messages |
| Straw *et al.* 2023 | - For scale-up, intervention should be led by the provincial Ministry of Health with involvement of primary health care worker authorities, community health care workers, Cervical Cancer prevention programmes, and the provincial Cancer Institute (political support and health authority involvement) - Human resources for HPV lab and Pap test lab at primary health care facilities are adequate to meet demand |
| Twimukye *et al*. 2021 | - Shorter turn-around time between receiving text and doctor calling (20-30 mins) - Promote peer support meeting (break stigma trends by sharing experiences and attaining peer mentorship) - Community sensitization about HIV and the intervention - Youth friendly HIV services and scale up app (add health spaces, designated space and staff for youth, resolve technical issues, supporting youth to overcome stigma and discrimination) |
| Venables *et al.* 2019 | - Initial text message upon enrolment in the intervention to know what to expect and understand how to respond to the intervention |
| Win Han Oo *et al.* 2021 | - Use both the electronic and paper-based system to kick-off intervention - Resolve technical challenges - Integrate policies and standard operating procedures for proper app operation - Plan at the beginning of the intervention how to sustain the intervention in the long term - Provide better quality devices (mobile phones, computers, tablets) that do not malfunction and IT support - Provide training for all stakeholders - Have a focal person who oversees managing the system at each level of leadership - Local experts to manage future upgrades, updates, system modification - Suggest “bring your own device” |

Abbreviations: HIV, human immunodeficiency virus; HPV, human papilloma virus; IT, information technology; SMS, short messaging service
